# Lithotrity—Statistics by M. Civiale

**Published:** 1847-03

**Authors:** 


*Lithotrity—Statistics by M. Civiale.*—From the year 1836 to 1845, M. Civiale has applied his method in 266 cases, with success in 259 patients, a few of which obtained only partial relief. In seventy-nine instances M. Civiale considered that the operation of crushing presented no chance of success, and refused to operate. Lithotomy was performed on twenty-eight, and seventeen recovered.

The statistics laid before the academy, at various periods, show that 582 patients have been operated upon by the author; and the tables point out distinctly this remarkable fact, that three-fourths of calculous patients who present themselves now-a-days, for treatment, are operated upon by the method of crushing.—***Lon. Med. Times.***

